# Enhancing the Validity of a Quality of Life Measure for Autistic People

**DOI:** 10.1007/s10803-017-3402-z

**Published:** 2017-11-29

**Authors:** Helen McConachie, David Mason, Jeremy R. Parr, Deborah Garland, Colin Wilson, Jacqui Rodgers

**Affiliations:** 1 Institute of Health and Society, Newcastle University, Newcastle upon Tyne, UK; 2 Institute of Neuroscience, Newcastle University, Newcastle upon Tyne, UK; 3 National Autistic Society Resource Centre, Newcastle upon Tyne, UK; 4 Autism Advocate, Sunderland, UK; 5 Institute of Health and Society, Newcastle University, Sir James Spence Institute level 3, Royal Victoria Infirmary, Newcastle upon Tyne, NE1 4LP UK

**Keywords:** Autism, Quality of life, Public mental health, Measurement properties

## Abstract

Accurate measurement of quality of life (QoL) is important for evaluation of autism services and trials of interventions. We undertook psychometric validation of the World Health Organisation measure—WHOQoL-BREF, examined construct validity of the WHO Disabilities module and developed nine additional autism-specific items (ASQoL) from extensive consultation with the autism community. The sample of 309 autistic people was recruited from the Adult Autism Spectrum Cohort-UK. The WHOQoL-BREF had good psychometric properties, including criterion, convergent, divergent and discriminant validity. The WHO Disabilities module showed adequate construct validity and reliability. The ASQoL items form a unitary factor of QoL, with one global item. Future studies can use the WHO measures alongside the ASQoL items to measure QoL of autistic people.

**Electronic supplementary material:**

The online version of this article (10.1007/s10803-017-3402-z) contains supplementary material, which is available to authorized users.

## Introduction

Measuring quality of life (QoL) of autistic people has been an under-researched area (Burgess and Gutstein 2007) but is gaining more empirical attention as awareness of the need to understand the lives of autistic adults increases (Chiang and Wineman 2014; Howlin and Magiati 2017; van Heijst and Geurts 2015). QoL is conceptualised as a multi-faceted construct that taps into different domains of life experience (Felce and Perry 1995; Harper 1998). Most studies have demonstrated that the QoL of autistic people is significantly lower than in general population samples (Jennes-Coussens et al. 2006; Kamp-Becker et al. 2010; Kamio et al. 2013) with a few exceptions (Hong et al. 2016; Moss et al. 2017).

It is important to validate existing measures and explore their psychometric properties with autistic people (Ikeda et al. 2014; Feldhaus 2015) so that the conclusions drawn from analyses are not called into question (Cottenceau et al. 2012). The measures used in studies of QoL of autistic people have not been specifically validated with autistic people (Ayres et al. 2017). Some have been validated for use with related populations (e.g. the comprehensive quality of life scale (Cummins 1997), which has parallel versions for the general population and those with intellectual disability). Mason and colleagues examined the validity of the WHOQoL (BREF) with a large sample (n = 370) of autistic adults and concluded that the original factor structure, as defined by the WHO, was adequate for use with autistic people (Mason et al. submitted-b). However, three items had loaded differently from those reported in the original structure (‘how well are you able to concentrate?’, ‘are you able to accept your bodily appearance?’, and ‘how well are you able to get around?’) and a mental health item did not load onto any factor (‘how often do you have negative feelings such as blue mood, despair, anxiety, depression?’). One explanation for this is that items were interpreted differently by autistic people from the original meaning; for example, the question about physical mobility was interpreted in terms of ease of getting around in the environment rather than referring to physical restrictions (Mason et al. submitted-b).

A lack of established measurement validity is a problem for QoL research with autistic people for three reasons. Firstly, it calls into question judgements about how to improve QoL for autistic people that are based on research using tools lacking established validity. For example, a recent study found that all WHOQoL-BREF domains are negatively predicted by having a mental health diagnosis and that some domains are positively predicted by receiving support and being in a relationship (Mason et al. submitted-a). If these, and other predictors (e.g. perceived informal support, Renty and Roeyers 2006; early diagnosis, Kamio et al. 2013; perceived stress, Hong et al. 2016, and social outcome, Moss et al. 2017) are used to inform targets for interventions for autistic people, the appropriateness of the targets is called into question if the outcome measure used in identifying the target is not sufficiently valid. Thus, a reliable and valid measure of QoL is required to measure progress and outcomes in intervention and treatment studies. Secondly, lack of a valid measure undermines comparisons of QoL with the general population (Jennes-Coussens et al. 2006; Kamp-Becker et al. 2010; Kamio et al. 2013; Mason et al. submitted-a) or other groups, for example people with Attention Deficit Hyperactivity Disorder, Disruptive Behaviour, or Affective Disorder (Barneveld et al. 2014). As such, the often reported lower QoL reported by autistic people may not actually reflect worse QoL but may reflect an unsuitable QoL measure. Finally, aspects of QoL may be interpreted differently by autistic people. Mason et al. (submitted-b) report that the mental health item of the WHOQoL-BREF was commented on by autistic people as conflating different feelings making it very difficult to answer. Similarly, an item about concentration (‘how well are you able to concentrate?’) was frequently interpreted in terms of the impact of sensory aspects of the environment (lights, noise, etc). These findings suggest that measurement of QoL may be subtly different for autistic people compared to the general population.

The WHOQoL suite of measures includes an additional module of items for people with intellectual or physical disability, the WHO Disabilities module (Power and Green 2010). This measure may have utility for capturing some important facets of QoL for autistic adults that are not addressed in the WHOQoL-BREF. It includes questions capturing perceptions of autonomy, discrimination and inclusion (Power and Green 2010); however, we expected that there would be some additional aspects of QoL salient to autistic people that are not captured by either WHO QoL measure such as sensory issues, e.g. hypersensitivity to sound, that are now a part of the diagnostic criteria for autism (American Psychiatric Association 2013).

### Aims of the Study

In order to enhance the validity of measurement of quality of life with autistic people, the present study had two linked objectives. The first was, with the participation of autistic adults, to develop autism-specific items to be used in conjunction with the WHOQoL-BREF and WHO Disabilities module to measure QoL. The second was to establish the convergent, divergent, discriminant, and construct validity; internal consistency; and test–retest reliability of the WHOQoL-BREF, WHO Disabilities module and autism-specific QoL items with autistic adults. The paper is structured in two stages for the separate objectives.

## Methods: Objective 1—Autism-Specific Item Development

### Summary of Process

**Fig. 1:**
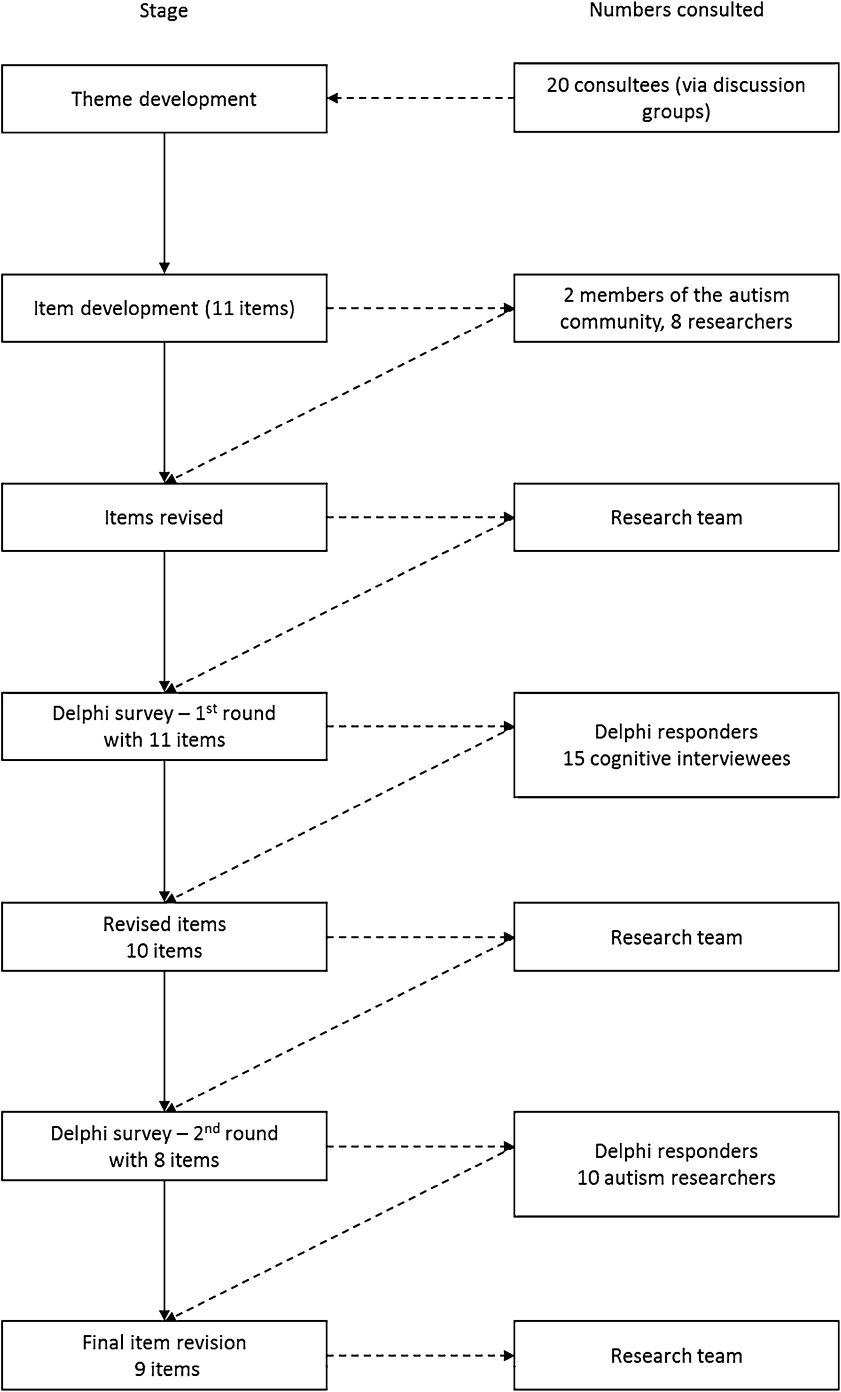
Flowchart for the item development stage of the study

A flow diagram showing the item development process can be found in Fig. 1. Four discussion groups were conducted with autistic people in the North East of England (Mason et al. submitted-b) considering the items included in the WHOQoL-BREF and WHO Disabilities module (see below). From the themes coded from the transcripts, items were developed to capture autism-relevant aspects of QoL not already covered. A Delphi survey and cognitive interviews were used to further refine the items, and autistic people and autism researchers were invited to comment on the draft autism-specific items. The final set of additional items was agreed by the authors.

### Consultation

Consultation with autistic people: Twenty autistic people were contacted via the National Autistic Society in the North East of England, and via a drama group for people with intellectual disability (Males = 13, Females = 7; mean age = 28.0 years, range 19–45). They attended one of four discussion groups which took place during summer 2016, led by a researcher, and two members of the autism community. As a group, consultees completed a systematic sorting task based on Q-sort methodology (Stephenson 1953) with the questions in the WHOQoL-BREF and Disabilities module. As well as the physical distribution of sorted questions into a pyramid shape of boxes according to importance, the process produced an ongoing ‘think-out-loud’ discussion. Second, consultees were asked to write down areas of QoL they thought were not covered by the WHOQoL-BREF or Disabilities items. Finally, consultees were shown the full WHOQoL-BREF/DIS questionnaire on paper, and the group discussed positive and negative views of the measure. Each person was thanked for their time and received a shopping voucher.

Consultation with Autism Researchers: On two occasions, at autism research meetings, researchers (n = 8 and n = 10) were presented with the draft autism-specific items and asked to comment on them. Researchers scored items on their importance and clarity of wording (see Delphi survey below for scoring scale). Researchers were also asked to review the terminology used for the draft items, highlight any concepts that may be difficult for autistic people to interpret and suggest alternative wording.

### Research Participants

Cognitive interviews: 15 autistic interviewees (9 men, 6 women; mean age 35.1 years, range 18 to 55) were recruited from the North East of England via the Adult Autism Spectrum Cohort-UK study (ASC-UK, http://research.ncl.ac.uk/adultautismspectrum/). This is a research programme about the life experiences of autistic adults and their relatives/carers. Each interviewee was thanked for their consultation and time with a shopping voucher.

Delphi survey: For the Delphi survey first round, 192 invitations were sent out to autistic participants via ASC-UK. These individuals had all previously completed the WHOQoL-BREF. Participants who could not give informed consent were also invited to take part, and were represented by a relative or carer authorised to act on their behalf (20 consented). There were 139 responders (72%; mean age 44.7 years, SD = 14.8). For the second round of the Delphi survey, 341 invitations were sent out and there were 235 responders (69%; mean age 42.8 years, SD = 14.2).

### Measures

#### WHOQoL-BREF (Harper 1998)

This is a 26 item measure that comprises two global questions and 4 QoL domains: Physical (7-items, e.g. ‘How well are you able to get around?’), Psychological (6-items, e.g. ‘To what extent do you feel your life to be meaningful?’), Social (3-items, e.g. ‘How satisfied are you with the support you get from your friends?’), and Environment (8-items, e.g. ‘How satisfied are you with your transport’). A higher score indicates a greater (better) subjective QoL. Participants complete the measure based on the two weeks prior to administration. Cronbach’s alpha demonstrates acceptable to good internal consistency for each domain: Physical QoL (0.82), Psychological QoL (0.81), Social QoL (0.68), and for Environment QoL (0.80) (Skevington et al. 2004).

#### WHO Disabilities Module (Power and Green 2010)

The Disabilities module was designed to be administered to people with physical or intellectual disabilities alongside either the WHOQoL-100 or WHOQoL-BREF. It has one global question (‘Does your disability have a negative (bad) effect on your day-to-day life?’). The other 12 items can be summed to give an overall QoL score, or scored in 3 domains: Discrimination (3-items, e.g. ‘Do you need someone to stand up for you when you have problems?’), Autonomy (3-items, e.g. ‘Do you feel in control of your life?’), and Inclusion (6-items, e.g. ‘Do you feel that your dreams, hopes, and wishes will happen?’). A higher score indicates greater subjective QoL. Participants complete the measure based on the 2 weeks prior to administration. Internal consistency has been shown to be good (Cronbach’s alpha = 0.85 for physical disability, and 0.80 for intellectual disability (Power and Green 2010)).

### Creating the Autism-Specific QoL Items (ASQoL)

In order to address the first objective (to develop some autism-specific items to be used in conjunction with the WHOQoL-BREF and Disabilities module), transcripts of each discussion group were examined for quotes that suggested either a nuanced understanding of a WHOQoL question, a direct suggestion for a new QoL item from a participant, or discussion that implied a missing aspect of QoL particular to the experiences of autism people. The transcripts were read through repeatedly by Authors 1 and 2; twelve themes were extracted and refined by all the discussion group leaders (see Supplementary material Table S1). Agreement for the coding of themes (i.e. relative frequency of application of codes throughout one transcript) of Authors 1 and 2 was calculated as 93.4%. On the basis of the themes, the authors drafted 11 potential items in similar format to the WHOQoL-BREF and Disabilities module questions. The items included concepts about barriers to accessing services, friendships, sources of support, and sensory issues.

### Cognitive Interview Schedule

Cognitive interviews were used to examine the understanding and appropriateness of the proposed autism-specific items. A semi-structured interview schedule consisting of standardised prompts was created to explore: (i) comprehension of the question, (ii) retrieval of relevant information, (iii) a judgement about retrieved information, and (iv) a response to the question that is intelligible (Boeije and Willis 2013). The last of these (iv) was assessed by asking the participant to complete the survey question. For example, the prompts for one item (‘Do sensory issues in the environment make it difficult to do things you want to do?’) were: (i) ‘How easy is the question to understand?’, (ii) ‘What does the question mean to you?’, (iii) ‘The question asks about sensory issues, what did you think about when you read the question?’ (iv) ‘How did you arrive at the answer you gave?’, and (v) ‘Is there anything else unclear about the question?’.

### Delphi Survey About Proposed Autism-Specific Items

Two rounds were designed for the Delphi survey, following methods used in research operationalising QoL for those with multiple disabilities (Petry et al. 2007). Participants completed the measures online (about 75%) or on paper. In round 1, the participant read the proposed QoL item and was asked, ‘How important is this question?’ responding with a five point Likert scale (not important, a little, moderately, very, and extremely important) and then ‘Is this question clearly worded?’ with a yes or no response. Results were collated; those items that fell below the threshold for consensus were presented in round 2. In addition, all comments were read through separately by Authors 1, 2 and 5, and then discussed together to inform further editing of the wording of items. In round 2, the participant was presented with the item from round 1, a short paragraph of text explaining why the item had been changed, and the amended version of the item. Participants then answered the questions about the item’s importance and clarity once more. For both rounds, there was free text space for comments about each item. A threshold of 80% consensus (i.e. 80% of participants rated the item as very or extremely important, and clear) was used for round 1, and 75% consensus for round 2 (Hasson et al. 2000).

### Procedure

Recruitment from ASC-UK: Participants in ASC-UK who had completed both the ASC-UK registration questionnaire (78 items including demographic information and the individual’s health and life situation) and the WHOQoL-BREF were contacted about the study. Participants living in the North East of England could consent to take part in the cognitive interview, Delphi survey, and/or psychometric validation portions of the study; participants living in the rest of England and Wales could consent to take part in the Delphi survey and/or psychometric validation. Potential participants were contacted by letter or e-mail along with a detailed information sheet, an abbreviated information sheet, and a consent form indicating the separate parts of the study.

Cognitive interview: Following receipt of the consent form, Author 2 made contact with the participant and arranged the interview at a time and place convenient to them. Seven interviews took place within research premises, seven took place at the participant’s home, and one in a quiet and private section of public space. Each interview was audio recorded and transcribed. The semi-structured interview schedule described above was followed for each participant. Cognitive interviews were used iteratively (Boeije and Willis 2013), with the proposed items being modified for the last 8 participants in light of revisions made following the first Delphi survey round.

Delphi survey: Following consent, participants received round 1 of the Delphi survey in the format of their choosing—either electronically (via Qualtrics), or in paper format. Additionally each participant was sent a list of all the items in the WHOQoL-BREF and Disabilities module in order to put the new ASQoL items into context. Participants who had completed round 1, and participants who returned their consent form subsequently, were sent round 2 of the survey. After each round, items that were above the threshold consensus were retained (some small changes to the wording were made if the cognitive interviews or survey free text comments suggested improvement was needed). Questions that did not meet the criteria were given more consideration by the research team, including autistic community representatives.

## Results: Objective 1—Autism-Specific Item Development

In Table 1 the final nine autism-specific (ASQoL) items are listed. Three of the items are negatively phrased, hence are reverse-scored. One of the items was intended as a ‘global’ QoL item about autistic identity.

**Table 1:** Finalised ASQoL items after two rounds of the Delphi survey and consultations

1. Do you have enough support from others to make important decisions? *For example, picking a course to study, finding a job, deciding where to live, planning for getting older*
2. Can you ‘be yourself’ around your friends/people you know well? *For example, you don’t have to put on an ‘act’*
3. How secure do you feel about your financial situation? *That is, that your current sources of income will continue (e.g. benefits, salary, pension etc.)*
4. Do you have enough support in your life, if or when you need it, to help you deal with problems? *For example, someone who knows you well and will give advice about social and other problems*
5. Are you satisfied with your current friendships? *(i.e. whether you have several, few, or no friends)*
^a^6. Do you feel there are barriers when accessing health services? *For example, staff do not allow you time to answer, or you cannot see the same GP*
^a^7. Do sensory issues in the environment make it difficult to do things you want to do? *For example, supermarket too noisy, public transport too busy, etc*
^a^8. Do you feel there are barriers to your needs being met in ‘official’ situations (e.g. at the benefit’s office, at work, with your landlord, etc.)? *For example, how other people communicate with you, or share information; feel unable to disclose your autism*
9G. Are you at ease (OK) with ‘Autism’ as an aspect of your identity? *Here, ‘Autism’ means any of the words that refer to the Autism Spectrum*

Items and scoring can be freely downloaded from http://research.ncl.ac.uk/cargo-ne/measures.html

^a^Indicates item is to be reverse scored; G indicates a global QoL item

In round 1 of the Delphi survey, items 2 and 7 (Table 1) were retained without change (importance consensus 80 and 82%; clarity consensus 96 and 98% respectively) (see Supplementary material Table S2). One item was discarded: ‘Do other people’s stereotyped expectations of autism have a negative impact on you?’ being rated by only 66% of respondents as ‘very important’ or ‘extremely important’. Furthermore, some participants commented that stereotypes may not be a negative issue if other people are unaware of an individual’s autism diagnosis. For example, “It is not relevant to me as I have not disclosed my autism to anyone except medical professionals”. The remaining eight items were modified based on the comments received and sent out to participants for round 2.

After round 2 of the survey, one further item was discarded due to a low importance rating (49%): ‘Do you feel able to help other people as much as you would like to?’. In addition, some participants noted the difference between having the capacity to help and having the opportunity to do so. They also commented on a lack of clarity regarding what ‘help’ might entail; for example, “Help can be interpersonal, involving personal interaction, or task-based (‘doing things for people’). Autistics can be good at the latter while being bad at the former”.

## Methods: Objective 2—Psychometric Evaluation

### Summary of Procedure

We aimed to assess:

1. the convergent, divergent, discriminant and criterion validity of the WHOQoL-BREF measure;
2. the construct validity, internal consistency, reliability and stability of the WHOQoL-BREF;
3. the factor structure, internal consistency, and reliability of the WHO Disabilities module;
4. the validity, internal consistency, and reliability of the new ASQoL items.

### Participants

Five hundred forty-four participants from ASC-UK were invited into the validation study. Of these, 426 participants consented to take part (78.3%) and a total of 309/426 completed the validation study (72.5%, 153 females (49.5%), 149 males (48.2%), and 5 (1.6%) who reported ‘other’ as their gender); two participants did not report gender. 31 participants (10.0%) reported they had help filling in the measures; 14 autistic people (4.5%) were represented by a relative or carer who completed the measures on their behalf; 260 participants (84.2%) reported they had no help completing the measures, and 4 participants (1.3%) did not answer the question.

When participants join the ASC-UK they provide information about what diagnosis they have, and who made the diagnosis. The mean age at diagnosis for the present sample was 37.35 years (SD = 16.10). The reported diagnoses were not verified by the research team. However, participants completed the Social Responsiveness Scale 2 (SRS2), a measure of autism severity (Constantino and Gruber 2012). A score of 52 is considered a cut-off for the presence of autism; the mean SRS2 total score for the sample was 110.35 (SD = 26.81).

### Measures

WHOQoL-BREF and WHO Disabilities module, ASQoL items: see descriptions above.

As the WHOQoL-BREF is scored as 4 domains (Physical, Psychological, Social, and Environment), a range of theoretically related *secondary measures* were included in the study for the assessment of validity.

#### Hospital Anxiety and Depression Scale (HADS) (Zigmond and Snaith 1983)

This is a 14-item measure of self-reported anxiety (7-items) and depression (7-items). Each item is scored between 0 and 3 with a higher score indicating more severe anxiety or depression. Depression and anxiety scores range from 0 to 21. Internal consistency is good for anxiety (anxiety, alpha = 0.80; depression, alpha = 0.76 (Mykletun et al. 2001) and for a young adult autistic sample: anxiety, alpha = 0.83; depression, alpha = 0.65 (Uljarevic et al. 2017)). For both anxiety and depression scales, scores of 0–7 indicate normal range, 8–10 is classified as ‘borderline’ suggesting the presence of depression/anxiety, and 11 or above indicates probable presence of depression/anxiety (Crawford et al. 2001; Snaith 2003). Participants complete the measure based on ‘how you have been feeling’ in the week prior to completion.

#### Craig Hospital Inventory of Environmental Factors-Short Form (CHIEF-SF) (Ephraim et al. 2006)

This 12 item measure comprises 5 subscales: policies, physical/structural, work/school, attitudes/support, and services/assistance designed to measure barriers faced by people with disabilities. Items are scored 0–4 (0, never; 1, less than monthly; 2, monthly; 3, weekly; and 4, daily). A higher score indicates a greater impact of environmental barriers. A ‘not applicable’ option is included for those not in work or school. Internal consistency is excellent (alpha = 0.92; (Liao et al. 2012)). Participants base their answers on their views from the year prior to completion.

#### Interpersonal Support Evaluation List-12 (ISEL-12) (Merz et al. 2014)

This measure contains 12 items which assess the perceived availability of social support in the general population. Each item is rated on a 4 point scale ranging from ‘definitely false’ to ‘definitely true’. Items are totalled to give a score between 0 and 36, with a higher score indicating higher perceived availability of social support. The measure has 3 subscales (each with 4 items; range of scores from 0 to 12): appraisal, belonging, and tangible. Internal consistency has been found to be acceptable or good for subscales (Cronbach’s alpha = 0.71 for the appraisal subscale; alpha = 0.76 for the belonging subscale; and alpha = 0.60 for the tangible subscale); however, the internal consistency for the overall measure has been found to be acceptable (alpha > 0.70) (Merz et al. 2014).

#### Comprehensive Quality of Life Questionnaire—Adult Version (ComQoL-A5) (Cummins 1997)

This measure includes two subjective QoL scales with one question per scale for each of the 7 QoL domains. Participants respond for each domain on a 5 point Likert scale (could not be more important, very, somewhat, slightly, and not at all important) and a 7 point Likert scale (delighted, pleased, mostly satisfied, mixed, mostly dissatisfied, unhappy, and terrible). The subjective QoL domains are: material well-being, health, productivity, intimacy, safety, community, and emotional well-being. Internal consistency for the satisfaction scale (alpha = 0.81) and the importance scale (alpha = 0.69) have been reported (Cummins 1997) for the general population.

### Procedure

Following consent, participants were sent the seven measures electronically (via Qualtrics) or via post as they preferred. Participants were asked to complete and return measures within 1 month. They received a brief description of each measure and information on the total number of items, and were invited to contact Author 2 if they had difficulties completing measures. The final question asked whether the participant had received assistance filling in the measures. Demographic data were available through the ASC-UK registration questionnaire (which records age, gender, mental health diagnoses, educational qualifications, etc). Participants were also asked to update their data about mental health diagnoses, hence the data reported on this are contemporaneous.

One month later, participants who had completed the measures online were re-sent three of the measures (the WHOQoL-BREF, WHO Disabilities module, and ASQoL items) in order to examine test–retest reliability.

A favourable ethical opinion for this study was granted by Wales REC 6 (reference—16/WA/0295) and by South Central—Oxford C REC (for including adults that are represented by a relative or carer; reference—16/SC/0598). A favourable ethical opinion for the ASC-UK study was granted by Wales Research Ethics Committee 5 (reference—14/WA/1066).

### Hypotheses and Analysis

Aim 1: To assess convergent and divergent validity, the specific hypotheses about relationships between the WHOQoL-BREF domains and secondary measures were: the HADS anxiety and depression scores will show the strongest correlations with the Psychological domain; the ISEL-12 score will correlate most strongly with the Social domain; the CHIEF-SF will correlate most strongly with the Environment domain. To assess discriminant validity, it was hypothesised that there would be a relationship between the HADS (cut-offs for presence of anxiety and depression) and lower scores on each domain of the WHOQoL-BREF. To assess criterion validity, it was hypothesised that each WHOQoL-BREF domain would correlate significantly with the ComQoL satisfaction subscale (and not necessarily with the importance subscale since WHOQoL-BREF measures satisfaction).

Aim 2: To assess the construct validity of the WHOQoL-BREF a confirmatory factor analysis was performed. Internal consistency was assessed with Cronbach’s alpha. Stability of scores was assessed at around a 12 month interval.

Aim 3: For the Disabilities module, an exploratory factor analysis was conducted to examine construct validity of the measure with autistic people.

Aim le 4: For the ASQoL items, an exploratory factor analysis was conducted to examine construct validity. Further validity analyses examined the correlations between the ASQoL total and global item scores, and WHOQoL-BREF domains.

For each WHO-related QoL measure, internal consistency was assessed using Cronbach’s alpha, and test–retest reliability via a second administration at approximately a 1 month interval.

#### Missing Data

Missing data percentages for the items in: WHOQoL-BREF ranged from 0.3 to 3.2%; WHO Disabilities module ranged from 0.6 to 3.9%; ASQOL ranged from 1.0 to 2.3%; ComQoL ranged from 0.6 to 1.3%; CHIEF-SF ranged from 1.0 to 4.9%; HADS ranged from 1.0 to 2.3%; and ISEL-12 ranged from 1.0 to 2.6%. The WHOQoL-BREF data cleaning process was applied to the data set. Subsequently, missing data were imputed for each measure using estimation maximisation as this is a robust method of imputation which outperforms mean substitution (Myers 2011). Participants that had more than 20% missing data were excluded (*n* = 3, leaving a sample of 306 for analysis).

## Results: Objective 2—Psychometric Evaluation

### Representativeness

Responders (n = 309) and non-responders (n = 117) were compared on the following data from ASC-UK registration: age, gender, Social Responsiveness Scale total (SRS2, a self-report measure of autism severity (Constantino and Gruber 2012)), and education level attained (highest formal qualification reported). T-tests were computed to assess group differences for age and SRS2 total and chi squared tests to assess differences in gender (male and female only) and qualifications (none and basic school leaver; advanced school leaver; degree level). Responders and non-responders did not differ on age (*t*(424) = − 0.728, *p* = 0.467, Cohen’s *d* = 0.08), gender (χ^2^(1, n = 417) = 0.268, *p* = 0.605, Cramer’s V = 0.25), SRS2 total score (*t*(350) = 0.113, *p* = 0.910, Cohen’s *d* = 0.01), or education qualifications (χ^2^(6, n = 417) = 0.964, *p* = 0.617, Cramer’s V = 0.05) (Table 2).

**Table 2:** Characteristics and demographic details of the validation sample (n = 309)

Age	Range	Mean age	Standard deviation
Age (years)	18–76	42.96	13.78
Male	18–76	45.71	14.92
Female	20–71	40.80	12.15

Age (by group)	*n*	Mean age	Standard deviation
16–25	42	22.69	1.83
26–40	96	32.86	3.93
41–60	141	50.69	5.62
61+	30	66.40	4.12

Demographics		*N*	%
Educational qualifications			
None		21	6.8
Basic school leaver		56	18.1
Advanced school leaver		85	27.5
Bachelor’s degree		58	18.8
Post graduate degree		60	19.4
Other/not reported		29	9.4
Current mental health condition diagnosis			
Yes		232	75.1
No		62	20.0
Not reported		15	4.9

A greater proportion of respondents preferred to complete the measures electronically (n = 231, 75%) rather than on paper (n = 75, 25%). It was expected that there might be demographic differences between those who completed online or on paper, and also those requiring help/not in completing the measures. Those who completed the measures online did not differ from those who completed paper versions on age (*t*(307) = 0.275, *p* = 0.784, Cohen’s *d* = 0.04) or SRS2 total (*t*(258) = 0.572, *p* = 0.568, Cohen’s *d* = 0.08). However, they did differ from online responders on qualifications attained (χ^2^(6, n = 309) = 15.183, *p* < 0.001; Cramer’s V = 0.225); those with lower level qualifications were more likely to complete the measures on paper. Those responding electronically were less likely to have help (χ^2^(1, n = 304) = 28.018, *p* < 0.001; Cramer’s V = 0.316). Relatives or carers acting on behalf of a participant lacking capacity to respond themselves (n = 14) were more likely to complete measures electronically.

### Descriptive Statistics

Table 3 shows the descriptive statistics for the WHOQoL-BREF and WHO Disabilities module transformed scores (ranging from 0 to 100); a higher score indicates better subjective QoL. For the ASQoL items the mean transformed score was 52.88 (SD = 12.32). Table 3 also shows comparisons with normative data for the WHOQoL-BREF (Skevington and McCrate 2012) and Disabilities module data (Power and Green 2010)^1^ and calculation of Cohen’s d effect size between the present sample and normative data for each domain. The effect sizes are large (except for Discrimination) and indicate that autistic people report lower quality of life. Table 3 also shows the proportion (and number) of participants within one standard deviation of normative data, more than one standard deviation below normative data, and more than one standard deviation above normative data. With the exception of the Environment and Discrimination domains, more than half of autistic people report their QoL to be more than one standard deviation below relevant norms. (Raw scores and Cronbach’s alpha are presented for the secondary measures CHIEF-SF, HADS, and ISEL-12, and transformed scores (0–100) for the ComQoL in Supplementary material Table S3.)

**Table 3:** Means and standard deviations for the present sample and normative data, with effect sizes. Proportions of the participants within, above, and below one standard deviation of normative values for the WHOQoL-BREF and WHO Disabilities module

Data source (n)	Physical	Psychological	Social	Environment
WHOQoL-BREF				
Validation data (306)	54.00 (22.74)*	43.89 (20.13)*	41.77 (22.82)*	56.19 (19.49)*
Normative (1324–1328)	76.49 (16.19)*	67.82 (15.56)*	70.52 (20.67)*	68.20 (13.81)*
Cohen’s d	1.14	1.33	1.32	0.71

	Discrimination	Autonomy	Inclusion	Total
Disabilities module				
Validation data (306)	45.15 (24.81)	58.82 (23.02)*	38.98 (21.94)*	45.49 (19.49)*
Normative (2561–2598)	43.67 (10.19)	66.25 (4.12)*	58.42 (9.90)*	56.88 (8.58)*
Cohen’s d	0.08	0.45	1.14	0.76

Proportion, % (n)	Physical	Psychological	Social	Environment
WHOQoL-BREF^a^				
>1 SD below norms	56.5 (173)	68.3 (209)	54.2 (166)	43.8 (134)
Within 1 SD of norms	37.9 (116)	29.7 (91)	44.8 (137)	48.4 (148)
>1 SD above norms	5.6 (17)	2.0 (6)	1.0 (3)	7.8 (24)

	Discrimination	Autonomy	Inclusion	Total
Disabilities module^b^				
>1 SD below norms	40.2 (123)	52.9 (162)	65.4 (200)	58.2 (178)
Within 1 SD of norms	22.9 (70)	14.4 (44)	23.9 (73)	24.8 (76)
>1 SD above norms	36.9 (113)	32.7 (100)	10.8 (33)	17.0 (52)

*Indicates a significant difference between the present sample and normative data, all *p* < 0.001

^a^Comparison values are taken from Skevington and McCrate (2012)

^b^Domain scores were computed by pooling item level mean, standard deviation, and sample size data taken from Power and Green (2010) before calculating proportions

### Psychometric Properties

#### Construct Validity—WHOQoL-BREF

An exploratory analysis of the factor structure of the WHOQoL-BREF suggested that the four domain model (Mason et al. submitted-b) was acceptable. Therefore, we conducted a confirmatory factor analysis with a new sample. Participants who had taken part in both the present study and the earlier study (Mason et al. submitted-b) were excluded from the CFA. This left 120 participants; this sample was augmented with a second sample of more recently recruited participants from the ASC-UK cohort study who had completed the WHOQoL-BREF, resulting in a final sample of N = 328 for the CFA, with a mean age of 38.49 (SD = 13.71; 142 males, 151 females, and 32 participants who recorded a gender of ‘other’ or did not report gender).

A CFA was conducted using 24 items (items 3–26; the two global items were excluded) to test the WHO factor structure (Harper 1998). Maximum Likelihood estimation was used and the overall fit was acceptable (CFI = 0.830, GFI = 0.852, RMSEA = 0.077, PCLOSE = 0.001, χ^2^ = 725.15, df = 246, *p* < 0.001). Modification indices were used to improve fit by covarying the error terms of items, starting with the highest modification index. After 4 iterations, the model fit had improved (CFI = 0.902, GFI = 0.886, RMSEA = 0.059, PCLOSE = 0.006, χ^2^ = 519.90, df = 242, *p* < 0.001) (see Supplementary material Fig. S1.) Thus the structural validity of the WHO factor structure is acceptable for use with autistic people.

#### Internal Consistency

Internal consistency was excellent for the overall WHOQoL-BREF measure (alpha = 0.93) comparable to UK population data (Skevington and McCrate 2012). Internal consistency was good for the physical (0.87), psychological (0.84), and environment (0.84) domains and acceptable for the Social domain (0.68).

### Convergent and Divergent Validity—WHOQoL-BREF

Pearson’s correlation coefficients were computed between WHOQoL-BREF domains and secondary measures for the hypotheses specified above; all tests were two-tailed (see Table 4). In addition, each correlation coefficient was transformed into a *z*-score to assess the comparative strength of correlations. The asymptotic covariance was estimated between each pair of correlation coefficients yielding an overall *z*-score and *p* value (Lee and Preacher 2013).

**Table 4:** Correlations between the WHOQoL-BREF domains and measures used to assess validity

	Physical	Psychological	Social	Environmental
Psychological QoL	0.670***	–		
Social QoL	0.326***	0.537***	–	
Environment QoL	0.713***	0.669***	0.418***	–
HADS depression	− 0.635***	− 0.756***	− 0.467***	− 0.590***
HADS anxiety	− 0.580***	− 0.600***	− 0.305***	− 0.539***
CHIEF-SF	− 0.670***	− 0.465***	− 0.265***	− 0.668***
ISEL-12	0.315***	0.455***	0.538***	0.500***
COMQOL importance	− 0.012	0.215***	0.115*	0.029
COMQOL satisfaction	0.682***	0.792***	0.590***	0.724***

Correlations in bold are those hypothesised to be strongest

*HADS* Hospital Anxiety and Depression Scale, *CHIEF-SF* Craig Hospital Inventory of Environmental Factors—Short Form, *ISEL-12* Interpersonal Support Evaluation List-12, *COMQOL* Comprehensive Quality of Life questionnaire—Adult version

*p < 0.05; **p < 0.01; ***p < 0.001

As hypothesised, the HADS Depression subscale was most strongly correlated with the Psychological domain, and the strength of correlation was significantly greater than that with the Physical domain (*z* = 3.985), Social domain (*z* = 7.502) and Environment domain (*z* = 5.306, all *p* < 0.001). The HADS Anxiety subscale was most strongly correlated with the Psychological domain; however, the strength of correlation was significantly greater only than that with the Social domain (*z* = 6.365, *p* < 0.001). As hypothesised, the ISEL-12 total score was most strongly correlated with the Social domain; however, the strength of correlation was significantly greater only than that with the Physical domain (*z* = 3.898, *p* < 0.001). Also as hypothesised, the CHIEF-SF was most strongly correlated with the Environment domain. That correlation was significantly stronger than for the CHIEF-SF with the Psychological domain (*z* = 5.629, *p* < 0.001) and the Social domain (*z* = 9.063, *p* < 0.001).

### Criterion and Discriminant Validity—WHOQoL-BREF

In terms of criterion validity, the ComQoL satisfaction subscale was, as hypothesised, significantly correlated with all WHOQoL-BREF QoL domains.

For assessing discriminant validity, WHOQoL-BREF domain scores were related to presence or absence of depression and anxiety. 3 × 4 MANOVAs were computed using the HADS cut-off values (normal range, borderline, presence). Level of severity of Depression had an overall significant effect on QoL domains (Wilk’s λ = 0.496, *F*(8,600) = 31.54, *p* < 0.001). Level of severity was a main effect on each domain of the WHOQoL-BREF (all *p* < 0.001) (see Table 5). Post hoc analyses showed that for Physical, Psychological, and Environment QoL each increase in HADS Depression severity resulted in significantly lower QoL. For Social QoL those with a categorisation of borderline depression had significantly lower QoL than those categorised as normal range but the difference between those categorised as borderline and those with probable presence of depression was non-significant (*p* = 0.243). Similarly, level of severity of Anxiety had an overall significant effect on QoL domains (Wilk’s λ = 0.691, *F*(8,600) = 15.24, *p* < 0.001). Level of severity was a main effect on each domain of the WHOQoL-BREF (Physical, Psychological, and Environment, *p* < 0.001; Social QoL, *p* = 0.001). Post hoc analyses showed that for Physical, Psychological, and Environment QoL each increase in HADS severity resulted in significantly lower QoL. For Social QoL those with a categorisation of presence of anxiety had significantly lower QoL than those categorised as normal range, but the difference between those categorised as borderline and those with probable presence of anxiety was non-significant (*p* = 0.61).

**Table 5:** Means and standard deviations for each QoL domain score related to HADS subscale score cut-offs. MANOVA effect sizes and post hoc comparisons are included

	WHOQoL-BREF domains			
	Physical QoL	Psychological QoL	Social QoL	Environment QoL
HADS depression				
Normal range	66.78 (19.92)*	57.62 (16.47)*	53.07 (19.96)^a,b^	65.63 (17.38)*
Borderline	53.25 (16.34)*	42.90 (12.42)*	36.17 (21.67)^a^	55.13 (15.85)*
Presence	37.94 (18.87)*	26.73 (13.79)*	30.49 (20.13)^b^	44.61 (17.66)*
Partial eta squared	**0.32**	**0.46**	**0.21**	**0.23**
HADS anxiety				
Normal range	76.88 (14.97)*	65.10 (17.20)*	51.24 (19.58)^a^	71.46 (15.32)*
Borderline	61.31 (20.39)*	48.80 (16.46)*	45.67 (22.11)	62.95 (15.45)*
Presence	46.40 (20.54)*	37.40 (17.84)*	38.36 (23.01)^a^	50.55 (18.92)*
Partial eta squared	**0.25**	**0.25**	0.05	**0.17**

For post hoc comparisons: for each domain score each matching superscript letter indicates significant difference for each pair of values labelled with the same letter

*Indicates all pairings significantly different

Partial eta squared = 0.01 for a small effect, 0.06 for a medium effect, and 0.13 for a large effect

### Construct Validity: Exploratory Factor Analysis Disabilities Module and ASQoL Items

Internal consistency for the Disabilities module was good (alpha = 0.89). Internal consistency for the Autonomy (0.78) and Inclusion (0.86) domains was good and was acceptable for the Discrimination domain (0.69).

Minimum rank factor analysis (MRFA) was used to examine the underlying factor structure of the Disabilities module and the ASQoL items (Lorenzo-Seva and Ferrando 2006). Polychoric correlations were used as this has been shown to outperform Pearson’s correlations when using ordinal data (Baglin 2014). Oblique rotation was selected to identify each factor model because each domain of the Disabilities module correlated significantly and it was anticipated that any separate factors arising from the ASQoL items would also be significantly correlated. Due to space restrictions only results for the final model of each factor analysis are outlined below (see Supplementary material Table S4 for factor loadings.)

#### Disabilities Module

Both Bartlett’s statistic (1809.3, *p* < 0.001) and the Kaiser–Meyer–Olkin (KMO) test (0.85) suggested good adequacy for the polychoric correlation matrix. For the three domain structure given by Power and Green (2010) a three factor solution emerged as the most robust and conceptually coherent. However, in this model three questions related to inclusion loaded with the three items of the discrimination scale. Both models explained a large proportion of the variance in the data: the one factor model yielded an explained common variance of 59.76% and this was 81.60% for the three factor model. Factor loadings were all strong, above 0.58 and 0.46 for the one factor model and three factor model respectively. The internal consistency of the one factor model was excellent (Cronbach’s alpha = 0.89). Internal consistency for each factor in this exploratory factor analysis was good to excellent: autonomy (0.78), inclusion (3 items; 0.80), and the combined items from discrimination and inclusion (0.83).

#### ASQoL Items

A one factor solution emerged as the most robust and coherent solution. Both Bartlett’s statistic (878.3, *p* < 0.001) and the Kaiser–Meyer–Olkin (KMO) test (0.82) suggested good adequacy for the polychoric correlation matrix. This model yielded an explained common variance of 66.6%. Item 9 (Are you at ease (OK) with ‘Autism’ as an aspect of your identity?) as expected did not load with the other items (loading = 0.18) and all other loadings were high (> 0.46). This suggested that item 9 can be conceptualised as a global QoL item. A second factor analysis was conducted on the eight items (excluding item 9) and this did not substantially change the results. Internal consistency for the eight items was excellent (Cronbach’s alpha = 0.82).

To evidence construct validity, the total ASQoL score (8 items) was used to predict the global QoL item. A regression analysis with total score as the dependent variable and the global item as the independent variable was significant (*R* ^2^ = 0.02, *p* < 0.01). Total score was a significant predictor of global ASQoL standardised (β = 0.15, *p* = 0.007).

Both the total score of the eight ASQoL items, and the global item, were correlated with the WHOQoL-BREF domains. The total score was significantly correlated with the Physical domain (*r*(306) = 0.67, *p* < 0.001), Psychological domain (*r*(306) = 0.67, *p* < 0.001), Social domain (*r*(306) = 0.53, *p* < 0.001), and Environment domain (*r*(306) = 0.79, *p* < 0.001).

#### Test–Retest Reliability

One hundred forty-one participants (46%) completed questionnaires at an interval of 3–5 weeks. Domain scores were calculated for each measure and intraclass correlation coefficient (ICC) estimates (along with 95% confidence intervals) were calculated. ICCs were based on a mean-rating (k = 2), absolute agreement, two-way mixed-effects model (Koo and Li 2016). The WHOQoL-BREF test–retest coefficients were: Physical domain ICC = 0.63 [0.47, 0.74], *p* < 0.001; Psychological domain ICC = 0.69 [0.54, 0.79], *p* < 0.001; Social domain ICC = 0.74 [0.64, 0.82], *p* < 0.001; and Environment domain ICC = 0.79 [0.71, 0.85], *p* < 0.001. The Disabilities module test–retest coefficients were: Total score ICC = 0.71 [0.61, 0.80], *p* < 0.001; Discrimination ICC = 0.74 [0.64, 0.82], *p* < 0.001; Autonomy ICC = 0.83 [0.76, 0.88], *p* < 0.001; and Inclusion ICC = 0.77 [0.68, 0.83], *p* < 0.001. The ASQoL test–retest coefficient for the total score was ICC = 0.76 [0.67, 0.83], *p* < 0.001.

### Stability—WHOQoL-BREF

As participants had initially completed the WHOQoL-BREF on joining ASC-UK, we estimated the stability of domain scores at about a 1 year interval; the gap between times of completion of the questionnaire was 8–21 months (mean = 13.30 months, SD = 3.3). In case having mental health difficulties might affect stability of QoL, the ICCs were calculated separately for 56 participants who did not report a mental health condition diagnosis at either time point, and 181 participants who did report a mental health condition at both time points. ICCs were, respectively: Physical domain ICC = 0.78 [0.62, 0.87] and 0.76 [0.68, 0.82]; Psychological domain ICC = 0.86 [0.76, 0.92] and 0.77 [0.69, 0.83]; Social domain ICC = 0.84 [0.73, 0.91] and 0.64 [0.52, 0.74]; and Environment domain ICC = 0.81 [0.74, 0.86] and 0.84 [0.73, 0.91]. All ICCs were significant at *p* < 0.001. Generally longer term stability of QoL scores was stronger than for 1 month test–retest reliability; Social QoL was less stable for those with ongoing mental health difficulties.

## Discussion

This study, involving a large cohort of autistic people across the adult age and ability range, is the first to present detailed validation of a measure of quality of life for this population. The appropriateness, reliability and structural validity of the internationally accepted WHOQoL-BREF along with the WHO Disabilities module have been demonstrated, as well as detailed comparison of WHOQoL-BREF domains with theoretically related measures to establish criterion, convergent, divergent and discriminant validity. Thus the findings of previous studies of autistic people that have utilised the WHOQoL-BREF can be generally accepted, that is, that quality of life of autistic people is significantly lower than for the general population.

An additional autism-specific module of nine items (ASQoL) was developed following extensive consultation with the autism community, covering issues such as sensory overload, friendships, barriers to accessing services, and identity as an autistic person. The items mostly relate to social factors (formal and informal acceptance and support), which may help to explain why the internal consistency of the WHOQoL-BREF Social domain is lower than for the other domains with this population. The structural validity and internal consistency of one global item and a total ASQoL score have been established. The ASQoL items are ready to be used by researchers (see Table 1 key), alongside the WHOQoL-BREF and WHO Disabilities module, to measure the QoL of autistic adults; this is an important step forward when considering evaluation of intervention trials and studies of the effectiveness of healthcare provision.

This is the first study to use the WHO Disabilities module (Power and Green 2010) with autistic people. It was notable that autistic people reported markedly lower inclusion in society than had physically disabled people in the normative study. The Disabilities module factor structure was partially replicated, though there were some different item loadings as three Inclusion items merged with Discrimination. One theme which arose from consultation with autistic people was experiencing a lack of respect and the problem of other people’s lack of knowledge about autism, which could contribute to both exclusion and perceived discrimination. A one factor solution emerged as optimal from the analysis, with excellent internal consistency.

One month test–retest reliability for most of the QoL domains was at the border of published criteria for moderate/good reliability (Koo and Li 2016); does this reflect a variable phenomenon or a not very reliable measure? As participants are asked to answer for the past 2 weeks, this may indicate that the measure taps into the current ‘state’ for autistic people rather than a more consistent ‘trait’. Indeed, particularly for the WHOQoL-BREF, some items capture what may be inherently variable facets of life over short spaces of time (e.g., sleep, concentration, safety). It is also the case that autistic people may not easily summarise information in order to generalise, given a particular style of ‘autistic sense-making’ (De Jaegher 2013); they are more likely to focus on detail or on what happened most recently. It is clear from the existing literature about autistic people’s lives (Elichaoff 2015; Hirvikoski and Blomqvist 2015; Bargiela et al. 2016; Hickey et al. 2017) that they encounter frequent and multiple stressors, and thus their perceived QoL (particularly physical and psychological) may indeed go up and down within a month’s interval. Stability over an average of 1 year was better for the WHOQoL-BREF domains, which suggests that the measure does have potential sensitivity as an outcome measure for evaluation of interventions. However, changes in QoL scores over a short period should perhaps be interpreted with caution. Certainly, the measure showed excellent discriminant validity in relation to whether autistic people have mental health difficulties.

The consultation with the autism community was multifaceted and thorough in developing the suggested new autism-specific module of items. A number of the research team’s expectations were confounded; for example, it was anticipated that ‘special interests’ would be mentioned as an important contributor to quality of life of autistic people (Jordan and Caldwell-Harris 2012), but that was not the case, neither in the discussion groups nor in the individual interviews where people talked freely within the activity structures. Furthermore, the global question introducing the WHO Disabilities module is ‘Does your disability have a negative (bad) effect on your day-to-day life?’; it was anticipated that autism described as a ‘condition’ would be preferred by participants, rather than ‘disability’, but no such comment was made during the study (Kenny et al. 2016). The ASQoL items will merit further study in other samples, in particular to explore more fully their face validity. There may also be important issues for autistic people (stress, adversity) not currently addressed which would merit future revision. For example, in the current study a number of participants talked about their roles as carers for others; however, the draft question ‘Do you feel able to help other people as much as you would like to?’ was judged not sufficiently important to retain from the Delphi survey, even though many comments were supportive regarding acknowledging autistic people’s positive contributions, not solely their problems.

### Strengths and Limitations

The study has a number of strengths including the large and varied sample of participants, with representation of people who needed help to complete questionnaires or for whom another person responded (15%). The sample was about half women, who are more usually under-represented in autism studies (Loomes et al. 2017). In future studies with the ASC-UK cohort, it would be useful to explore the reasons why women may be more willing to volunteer to take part in studies of their life-experiences, and also to examine the equivalence of completion by paper or online format, as has been demonstrated for other questionnaires in both disabled and general populations (Weigold et al. 2013; Bagby et al. 2014; Bishop et al. 2010).

The confirmatory factor analysis of the WHOQoL-BREF data was carried out with an independent sample of autistic people, building on a previous exploratory analysis (Mason et al. submitted-b), providing evidence that the measure is robust for use with autistic people. One limitation is that the diagnoses of the participants were not verified by the research team; however, the mean SRS2 score is approximately double the cut-off for autism on the measure. Furthermore, the level of functioning and IQ could be important characteristics to consider when validating measures. In the present sample, a large proportion had achieved advanced school leaver qualifications or higher (approximately 66%) and this suggests a cognitively able sample. As such, it would be useful for future studies looking at the validity of the WHOQoL-BREF with autistic people to assess in greater detail diagnoses, functioning, and cognitive ability to explore the generalisability of findings.

The consultation process with autistic people was extensive; nevertheless it did not start from an open-ended enquiry about what matters for people in terms of quality of life, which might have elicited additional themes, nor has the consultation as yet involved autistic people from countries other than the UK. The eight ASQoL items accounted for just 2% of the variance in the global item. Although a significant predictor it does suggest that these items capture only a small aspect of ‘autistic identity’. Future work into other identity domains (McDonald 2017) and the ASQoL would expand understanding of autistic identity further. Through data sharing and use with other large samples, the adequacy and strengths of the ASQoL module can be tested in future.

### Implications

The WHOQoL measures along with the newly developed ASQoL show promise for use with autistic people in clinical settings to elucidate some of the challenging issues they may be experiencing in their lives. The new items pick up on concerns such as sensory overload, lack of financial security and barriers to accessing healthcare that may affect a broad range of people but which are particularly salient for autistic people. Quality of life is clearly reduced by depression and anxiety but accurate measurement in studies will require use of an additional measure for mental health, as there is only one such question in the WHOQoL-BREF (Mason et al. submitted-b). Given the poor performance of the mental health item (0.18 loading in the CFA), further consultation could explore whether to include mental health items that are relevant to autistic people in the ASQoL, or instead whether a separate mental health screener is preferable. Mental health issues are very common in autistic people (Lever and Geurts 2016; Russell et al. 2016) and yet intervention and support services with staff knowledgeable about autism are still insufficient. Future research will need to establish the sensitivity of the combined measures in evaluation of new services and interventions (Bishop-Fitzpatrick et al. 2014) but the current study to validate a quality of life measure with autistic adults is an important step forward.

## Electronic supplementary material

Below is the link to the electronic supplementary material.

Supplementary material 1 (DOCX 167 KB)

## Footnotes

1 The normative data presented for the Disabilities module are mean scores and standard deviations for people with physical disabilities (Power and Green 2010). Mean domain scores were computed by taking the mean of each item belonging to that domain. Standard deviations were computed by first finding pooled variance using following steps: multiplying the sample size of the item by the variance of that item; multiplying the sample size of each item by the difference between the item and domain mean the item belongs to; summing the previous two steps together for each item in the domain; and dividing the result by the total sample size of each item in the domain minus one. The square root of the pooled variance was then calculated to yield a standard deviation for each domain.
