# Supplementary material for: Enhancing the Validity of a Quality of Life Measure for Autistic People
Source: J Autism Dev Disord. 2017 Nov 29;48(5):1596–611. doi: 10.1007/s10803-017-3402-z (PMC5889785; doi:10.1007/s10803-017-3402-z)

**Supplementary material**

**Manuscript title: Enhancing the validity of a Quality of Life measure for autistic people**

Journal: Journal of Autism and Developmental Disorders

Helen McConachie^1^, David Mason^1^, Jeremy R Parr^2^, Deborah Garland^3^, Colin Wilson^4^, Jacqui Rodgers^2^

1 Institute of Health and Society, Newcastle University, UK

2 Institute of Neuroscience, Newcastle University, UK

3 National Autistic Society Resource Centre, Newcastle upon Tyne, UK

4 Autism advocate, UK

Corresponding author: Professor Helen McConachie; [helen.mcconachie@ncl.ac.uk](mailto:helen.mcconachie@ncl.ac.uk)

**Table S1: Discussion group coding of themes.**

The coding categories pull out of the transcripts aspects of the discussion which are about what may be **different for autistic people** and their experiences. The aim of the coding is to suggest important factors in quality of life that are missing from the existing questionnaires and might form the basis for new items.

Statements that are simply how participants would have answered the item for themselves, nor how important they thought the item to be, were not coded.

|  | Code: | Description: |
| --- | --- | --- |
| 1 | Autism related knowledge | Other people’s lack of knowledge about autism - societal views, individual people encountered, employers, professionals (i.e. medical). This includes the adaptations to procedures that autistic individuals would like or which have been made. |
| 2 | External support and services – access and barriers | Reference to gatekeeping / gatekeeping culture. Issues of trust in people providing services. Loss of support, either financial or the people / organisations that support the individual. Someone in an official capacity who will stand up for the individual. Need for autism-specific service. |
| 3 | Family support | The role family play in the life of an adult with autism; support could be social, monetary, or advocacy. This relates to parents, partners, spouses etc. |
| 4 | Sensory issues | Issues around distractibility, loud noise etc. (i.e. interaction with the environment) that may have an impact on autistic individuals. |
| 5 | Internal characteristics of autism | Ways that people generally do things that may not be adaptive. Issues that may prevent autistic people from obtaining jobs/resources etc. Examples include protracted worrying about the past, or future possibilities; stress in many situations. Self-knowledge of one’s limitations or abilities/skills. |
| 6 | ‘Autistic’ identity | The sense of identity that ‘autism’ provides and the opportunities to socialise with other autistic people. |
| 7 | Autonomy, self-determination | Points related to having a desire (or lack of desire) about decision making, whether decision making is something that is always a good. Choosing how to live; enjoying being on one’s own. |
| 8 | Mental health issues | Experiences of high anxiety, depression, etc, and the circumstances that may lead to this. Consequences of feeling depressed/anxious. (nb. Discussion of more than everyday stress.) |
| 9 | The nature of friendship | What people want friends for; whether they want friends |
| 10 | Social engagement | Comments about social interaction being difficult for an autistic adult, or tiring, or not feeling accepted, or issues about safety. |
| 11 | Appropriate local activities | Whether the individual feels able to take part in society because activities are not suitable, or other reasons why community participation is not sought out. |
| 12 | Suggested items | Possible items directly suggested by participants |

**Table S2: Delphi Survey items**

Threshold criteria: After round 1 of the survey, two items (in bold) were retained having been rated as both important and clear by at least 80% of the participants and judged as requiring only minor wording adjustment; these were not re-presented in round 2. For round 2 the threshold for retaining an item was 70% for both importance and clarity.

| Mean ratings of importance and clarity for each item in Delphi round 1 and round 2. | | |
| --- | --- | --- |
| Item | Importance  (%) | Clarity  (%) |
| Round 1 – 11 items |  |  |
| Do other people’s stereotyped expectations of autism have a negative impact on you? | 66 | 92 |
| Do you have enough support in your life to make important decisions? | 79 | 96 |
| **Can you ‘be yourself’ around your friends/people you know well?** | **80** | **96** |
| Do you have enough support in your life to help you to avoid problems? | 90 | 90 |
| How satisfied are you with your friendships (or are you satisfied without friendships)? | 70 | 87 |
| How satisfied are you with your capacity to be helpful to other people? | 50 | 96 |
| How satisfied are you with your current financial security? | 80 | 98 |
| Do people in the health services (e.g. GP, dentist, nurse) make some adjustment to meet your needs? | 78 | 92 |
| **How often do sensory issues in the environment make it difficult to do things you want?** | **82** | **98** |
| Do you feel that people in ‘official’ situations make adjustments to meet your needs? | 84 | 92 |
| Are you at ease (OK) with your identity as an ‘autistic’ person? | 80.5 | 93.2 |
| Round 2 – 8 items |  |  |
| Do you have enough support from others to make important decisions?  *For example, picking a course to study, finding a job, deciding where to live, planning for getting older.* | 80 | 95 |
| Do you have enough support in your life, if/when you need it, to help you deal with problems?  *For example, someone who knows you well and will give advice about social and other problems.* | 84 | 88 |
| Are you satisfied with your current friendship situation?  *That is, whether you have several, few, or no friends* | 74 | 90 |
| Do you feel able to help other people as much as you would like to?  *For example, give support or advice to a member of your family, to other autistic people, etc.* | 49 | 92 |
| How secure do you feel about your financial situation?  *That is, that your current sources of income will continue (e.g. benefits, salary, pension etc.)* | 88 | 94 |
| Are there barriers to your needs being met when accessing health services?  *For example, staff do not allow you time to answer or explain treatments clearly, you cannot see the same GP* | 82 | 78 |
| Do you feel there are barriers to your needs being met in ‘official’ situations (e.g. benefits office, work, housing provider, etc.)?  *For example, how other people communicate with you, or share information; feel unable to disclose your autism diagnosis* | 85 | 76 |
| Are you at ease (OK) with ‘autism’ as an aspect of your identity? | 71 | 88 |

**Table S3: Descriptive statistics for additional measures used in the study**

The table below shows the mean score and standard deviations for each of the additional measures used to validate the WHOQoL-BREF. The CHIEF-SF ranges from 0 to 48. Please note, transformed scores (i.e. a 0 to 100 scale) are presented for the COMQOL as indicated in the scoring manual.

| **Table S3: Means (and standard deviations) for secondary measures used in the validation study** | | | | | |
| --- | --- | --- | --- | --- | --- |
| Measure | Mean | | Standard deviation (SD) | | Cronbach’s alpha |
| CHIEF-SF | 16.61 | | (9.84) | | 0.88 |
| HADS |  | |  | |  |
| Depression | 8.52 | | (4.76) | | 0.83 |
| Anxiety | 12.48 | | (4.59) | | 0.86 |
| ISEL-12 |  | |  | |  |
| Total score | 18.18 | | (7.12) | | 0.88 |
| Appraisal | 6.54 | | (3.03) | | 0.81 |
| Belonging | 4.80 | | (2.46) | | 0.71 |
| Tangible | 6.84 | | (2.93) | | 0.77 |
| COMQOL | Importance | | Satisfaction | |  |
|  | Mean | SD | Mean | SD |  |
| Total QoL | 63.84 | (14.61) | 53.88 | (17.53) |  |
| Material well-being | 64.13 | (21.56) | 72.59 | (17.27) |  |
| Health | 70.90 | (23.07) | 46.24 | (26.15) |  |
| Productivity | 61.97 | (27.16) | 48.10 | (26.47) |  |
| Intimacy | 68.61 | (26.24) | 57.62 | (24.47) |  |
| Safety | 73.03 | (22.37) | 57.31 | (25.68) |  |
| Place in community | 41.45 | (27.85) | 48.72 | (23.67) |  |
| Emotional well-being | 66.82 | (24.35) | 46.57 | (25.42) |  |
| Cronbach’s alpha | 0.69 | | 0.85 | |  |

**Table S4: Factor analysis of the WHO Disabilities module and ASQoL**

For each factor analysis a three step procedure was followed. Step 1: Bartlett’s statistic and sampling adequacy were inspected and a minimum factor loading cut-off of 0.30 was chosen for item inclusion (due to a sample size greater than 300 will yield a significant item load of 0.32 with an alpha of 0.001; Yong & Pearce 2013). Step 2: the original unitary model of the WHO Disabilities module structure was tested but the measure can be employed with 3 domains (discrimination, autonomy, and inclusion, Power & Green 2010). The ASQoL items were tested with no a priori structure except an expectation that the autistic identity question (the global QoL item) would not load onto the factor model. The WHO Disabilities module was tested for a unitary structure and with a two, three, and four factor model in line with good exploratory factor analysis practice (Osborne & Costello 2009) and the ASQoL items were tested with a one and two factor model. Step 3: internal consistency (Cronbach’s alpha) was computed for each domain.

| Factor loadings for the WHO Disabilities module and ASQOL items. | | | | | |
| --- | --- | --- | --- | --- | --- |
|  |  | WHO Disabilities module | | | |
|  |  | One factor | Three factors | | |
|  |  |  | Aut. | Inc. | Disc. and Inc. |
| 2 | Do you feel that some people treat you unfairly? | 0.65 | -0.08 | 0.05 | **0.75** |
| 3 | Do you need someone to stand up for you when you have problems? | 0.62 | 0.39 | -0.36 | **0.71** |
| 4 | Do you worry about what might happen to you in the future? | 0.59 | 0.02 | 0.12 | **0.56** |
| 5 | Do you feel in control of your life?  For example, do you feel in charge of your life | 0.74 | **0.46** | 0.25 | 0.29 |
| 6 | Do you make your own choices about your day-to-day life?  For example, where to go, what to do, what to eat. | 0.62 | **0.88** | 0.07 | -0.03 |
| 7 | Do you get to make the big decisions in your life?  For example, like deciding where to live, or who to live with, how to spend your money. | 0.58 | **0.86** | 0.07 | -0.05 |
| 8 | *Are you satisfied with your ability to communicate with other people?* | 0.72 | 0.25 | 0.19 | **0.47** |
| 9 | *Do you feel that other people accept you?* | 0.81 | -0.14 | 0.24 | **0.84** |
| 10 | *Do you feel that other people respect you?* | 0.78 | 0.03 | 0.31 | **0.61** |
| 11 | Are you satisfied with your chances to be involved in social activities? | 0.74 | 0.03 | **0.86** | 0.12 |
| 12 | Are you satisfied with your chances to be involved in local activities? | 0.72 | 0.20 | **0.75** | 0.05 |
| 13 | Do you feel that your dreams, hopes and wishes will happen? | 0.75 | 0.28 | **0.49** | 0.23 |
|  | | ASQoL |  |  |  |
| 1 | Do you have enough support from others to make important decisions? | 0.79 |  |  |  |
| 2 | Can you ‘be yourself’ around your friends/people you know well? | 0.65 |  |  |  |
| 3 | How secure do you feel about your financial situation? | 0.56 |  |  |  |
| 4 | Do you have enough support in your life, if or when you need it, to help you deal with problems? | 0.81 |  |  |  |
| 5 | Are you satisfied with your current friendships? | 0.46 |  |  |  |
| 6 | Do you feel there are barriers when accessing health services? | 0.69 |  |  |  |
| 7 | Do sensory issues in the environment make it difficult to do things you want to do? | 0.59 |  |  |  |
| 8 | Do you feel there are barriers to your needs being met in ‘official’ situations (e.g. benefit’s office, at work, your landlord, etc.)? | 0.72 |  |  |  |
| 9 | Are you at ease (OK) with ‘Autism’ as an aspect of your identity? | 0.18 |  |  |  |
| Aut. = Autonomy; Inc. = Inclusion; Disc. = Discrimination.  NB Items in italics do not load as expected from Disabilities module structure. Bold numbers indicate strongest factor loading per item (not shown for the one factor solutions; ASQoL item 9 did not load). Some items (i.e. Disabilities module items 4 and 8, ASQoL items 1 and 2) have examples that accompany the item. These have not been included for reasons of space. | | | | | |

**Figure S1: Confirmatory factor analysis (CFA) for the WHOQoL-BREF**


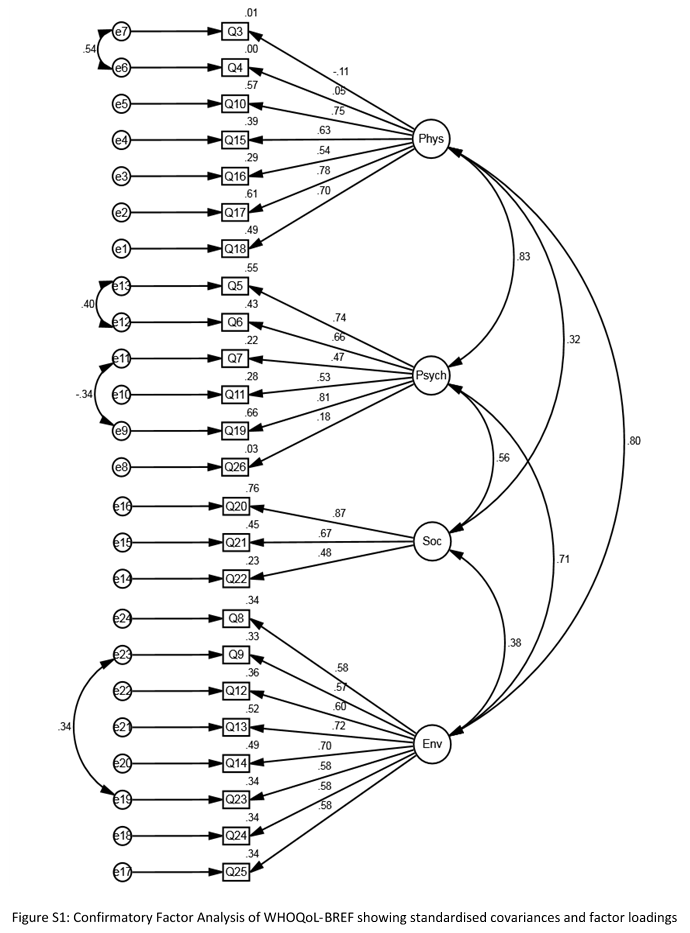

Supplement: Supplementary file 1 — Supplementary material 1 (DOCX 167 KB) [file 10803_2017_3402_MOESM1_ESM.docx]
